# Improvement of polydopamine-loaded salidroside on osseointegration of titanium implants

**DOI:** 10.1186/s13020-022-00569-9

**Published:** 2022-02-21

**Authors:** Qingqing Yi, Pengchen Liang, Dongyu Liang, Liou Cao, Shuang Sha, Xinquan Jiang, Qing Chang

**Affiliations:** 1 Clinical Research Center, Jiading District Central Hospital Affiliated Shanghai University of Medicine & Health Sciences, Shanghai, 201800 China; 2 School of Microelectronics, Shanghai University, Shanghai, 201800 China; 3 Shanghai Key Laboratory for Molecular Imaging, Shanghai University of Medicine and Health Sciences, Shanghai, 201318 China; 4 Department of Prosthodontics, Shanghai Engineering Research Center of Advanced Dental Technology and Materials, Shanghai Key Laboratory of Stomatology & Shanghai Research Institute of Stomatology, National Clinical Research Center for Oral Diseases, Shanghai Ninth People’s Hospital, College of Stomatology, Shanghai Jiao Tong University School of Medicine, Shanghai, 200011 China

**Keywords:** Titanium, Microarc oxidation, Polydopamine, Salidroside, Osteogenesis, Angiogenesis

## Abstract

**Background:**

Microarc oxidation (MAO) on the surface of medical pure titanium can improve its histocompatibility, and loading drugs on the surface can resist excessive intimal hyperplasia.

**Methods:**

In this study, salidroside (SAL) was loaded on the surface of porous titanium (Ti) with polydopamine (PDA) carrier. The effects of SAL on the osteogenesis and angiogenesis of Ti implants were studied by phalloidin staining, alizarin red staining, ALP staining, wound-healing assay, cell transwell assay, matrigel tube formation, and osteogenic and angiogenic genes and proteins expression detected by PCR and western blot in vitro. The bone defect model experiments in rats was established in vivo including X-ray, micro CT, hematoxylin and eosin staining (HE), immunohistochemistry (IHC), Goldner's trichrome analysis, Safranin O-fast green staining and determination of contents of TNF-α and IL-6 in serum.

**Results:**

EDS and EDS mapping showed that SAL could be loaded on the surface of the MAO coating by PDA. A drug release experiment showed that SAL loaded on the Ti coating could release slowly and stably without sudden release risk. In vitro cell experiments showed that the SAL coating could promote the proliferation, morphology, calcification and alkaline phosphate activity of MC3T3-E1 cells. At the same time, it promoted the migration and tube formation of HUVEC cells. The SAL coating promoted osteogenesis and angiogenesis by promoting the expression of genes and proteins related to. In vivo experiments, HE and IHC showed that SAL significantly promoted the expression of COL-1 and CD31. Goldner's trichrome and Safranin O-fast green staining showed that SAL coating could increase the new bone tissue around the implantation site. The SAL coating had anti-inflammatory activity by reducing the levels of TNF-α and IL-6 in vivo.

**Conclusion:**

Therefore, SAL could improve osteogenesis and angiogenesis in conjunction with the Ti-PDA coating.

## Introduction

Single-metal biomedical materials have several major problems in clinical application. In the physiological environment, the surface of metal implants quickly becomes corroded, leading to the increase of wear debris and the release of metal ions, which causes damage to the body. At the same time, the implant surface lacks antibacterial ability, which makes the implant prone to bacterial infection and may lead to serious postoperative infection complications. In addition, owing to the poor biocompatibility of the metal implant surface, the bone conductivity and integration of the implant are reduced, resulting in poor fixation of the implant in vivo and increasing the risk of secondary surgery [1]. To overcome the problems caused by surface defects of metal implants, various types of surface modifications (involving chemical, physical and biological technologies) can be conducted on implants to improve the biocompatibility and antimicrobial properties of the implant surface so as to promote bone healing at the early stage of implantation and reduce the related risks of implantation infection [2].

MAO is a surface treatment technology used to form a ceramic coating on Ti, aluminum, magnesium and other metals. Through MAO treatment, some bioactive elements can be incorporated into the surface of metal implants to give them a nanoporous structure, which improves the biological and functional characteristics of implants and makes them more feasible for clinical use [3, 4]. Hydroxyapatite, the main component of bone tissue, can be used to improve the biocompatibility between implant and bone tissue. Therefore, it has been widely used in the biomedical field for the past 10 years [5]. However, the antibacterial activity, biocompatibility and osteogenic activity of MAO implants are still poor, so the surface needs to be further modified. PDA is similar to mussel protein in structure and has good biocompatibility and bioactivity [6, 7]. PDA can form a film on almost any material, and it is widely used in drug loading, adsorption and antibacterial applications.

Clinical osteogenic drugs can be divided into those used in Western medicine and those used in traditional Chinese medicine. Osteogenic drugs used in traditional Chinese medicine have few side effects and produce little drug resistance in long-term application, which shows obvious advantages in the research and development of new osteogenic drugs. Previous studies have shown that catalpol [8], icariin [9], resveratrol [10] and salidroside [11] used in traditional Chinese medicine have bone promoting activity. SAL, chemical name 4-hydroxyphenyl-β-d-glucopyranoside, is a phenolic glycoside extracted from the roots and stems of Rhodiola plants [12]. SAL has cardiological, cerebral and vascular protection, immune regulation, anti-tumor, osteogenesis and other pharmacological effects [13].

SAL has been confirmed osseointegration and anti-inflammatory activities used in the treatment of a variety of diseases, such as bone defect, fracture, arthritis, etc. [14]. The mechanism may be related to the inhibition of NF-κB phosphorylation by SAL, which affects a series of related signaling pathways. Some scholars used the osteoarthritis rats induced by anterior cruciate ligament transection as the experimental model, and found that SAL could inhibit the phosphorylation of NF-κB, reduce the expression of TNF-α, VCAM, MMP-13 and IL-17, and promote the production of intracellular collagen and glycans. Thereby, it promotes the proliferation of chondrocytes,and has a certain preventive and therapeutic effect on osteoporosis and cartilage degeneration.

At present, SAL is administered orally or intravenously. However, neither oral nor intravenous administration of traditional Chinese medicine extracts or monomers can produce a sustained high concentration in the microenvironment in the lesion area, so the local treatment effect is poor. Coating SAL on the surface of metal biomedical materials could enable local administration, which offers the advantages of high concentration, strong effect, small dose, short course of treatment and light side effects. Therefore, a Ti calcium phosphorus SAL composite film was prepared in this study. The effects of SAL on bone regeneration and angiogenesis after bone defect and the mechanisms of these effects were further studied.

## Materials and methods

### Materials preparation and characterization

Pure medical grade Ti was cut into 10 mm × 10 mm × 1 mm pieces and then polished with sandpaper until the surface was smooth and traceless. The Ti plate was put into anhydrous ethanol for ultrasonic cleaning for 5 min and rinsed with distilled water three times. The pure Ti specimens were immersed in an electrolyte solution composed of 5 g/L sodium hexametaphosphate ((NaPO_3_)_6_, RHAWN, Shanghai, China), 12 g/L sodium silicate (Na_2_O·nSiO_2_, RHAWN, Shanghai, China), 3 g/L sodium hydroxide (NaOH, RHAWN, Shanghai, China) and 2 g/L glycerol (C3H_8_O_3_, RHAWN, Shanghai, China) for MAO treatment. MAO equipment (Micro Arc Environmental Protection Company, Dongguan, China) with a 70 kW bipolar pulse MAO power supply, current 3 A/dm^2^, frequency 600 Hz, duty cycle 25% and processing time 5 min.

After MAO, the Ti plate was put into anhydrous ethanol for ultrasonic cleaning for 10 min and washed with distilled water three times. After natural drying, it was put into a 3 mol/L NaOH aqueous solution in a water bath at 60 °C for 1 h for alkali activation treatment. The test specimen was put into 5 mg/mL dopamine in Tris HCl (pH = 8.5) buffer solution and soaked in the dark for 12 h. The dried samples were immersed in 0 mg/mL, 1 mg/mL and 2 mg/mL SAL (Aladdin, Shanghai, China) PBS solution for 12 h. The samples were then rinsed with distilled water three times and dried naturally before use. The quality difference of the coating before and after loading PDA and SAL was weighed by microgram precision balance to determine the loading amount of PDA and SAL on the coating. Four different groups-uncoated (Ti) and coated (Ti-PDA, 1 mg SAL-PDA and 2 mg SAL-PDA)-were compared.

The surface morphology of each coating was observed with a field emission scanning electron microscope (SEM, SU8010, Hitachi, Japan), and the element composition and distribution of the coating were analyzed by energy dispersive spectrometer (EDS, X-MAX 150, Oxford, UK).

### In vitro experiments

#### Release of SAL

To test the release of SAL, first 100 mg SAL was dissolved in a mixed solvent of acetonitrile and H_2_O (volume ratio 1:1) with a volume of 10 mL. Then the solution was transferred to a volumetric flask to prepare a series of solutions with concentrations of 0.125, 0.25, 0.5, 1, 1.5, 2 and 2.5 mg/mL. The absorbance of each solution at 275 nm was measured to draw its A-C curve.

The SAL-loaded composite film was placed in a 30 mL degradation bottle with 20 mL Hank's simulated body fluid, and the static release experiment in vitro was conducted in a 37 °C water bath. On the 5th, 13th, 21st and 30th days, 3 mL solution was taken out and mixed with 1 mL dichloromethane. The mixture was centrifuged at 4000 rpm for 5 min. Then, the lower layer was taken out and mixed with 4 mL medium (acetonitrile and water with a 1:1 volume ratio). The results were analyzed by an ultraviolet spectrometer (ULS4096CL, AvaSpec, Netherlands) after shaking evenly and filtrating. The concentration was calculated by fitting equation, and the release rate was obtained.

#### Culture of cells

Mouse embryo osteoblast precursor cells (MC3T3-E1) were cultured in dulbecco's modified eagle medium (DMEM, HyClone, Logan, UT, USA), and human umbilical vein endothelial cells (HUVECs) were cultured in α minimum essential medium (α-MEM, HyClone, Logan, UT, USA) supplemented with 10% fetal bovine serum (FBS, Gibco, Thermo Fisher Scientific, Waltham, MA, USA), 100 U/mL penicillin and 100 μg/mL streptomycin.

#### Morphological observation of cytoskeleton

The pure Ti, Ti-PDA, 1 mg SAL-PDA and 2 mg SAL-PDA were put into 24-well plates, and MC3T3-E1 cells were inoculated to the surface of each specimen with 5 × 10^4^ cells/well. The cells were cultured in an incubator for 5 days. Five-day cell cultures were fixed in 0.5 ml of 4% paraformaldehyde for 30 min. Then, cells were stained with 5 μg/mL of phalloidin (Solarbio, Beijing, China) for 60 min. Next, cells were stained with 200 μl of DAPI (Solarbio, Beijing, China) for 10 min. Finally, the morphology of cytoskeleton was observed under microscope after PBS washing.

#### Alizarin red staining

MC3T3-E1 cells were cultured with coatings in 24-well plates for 24 h. Osteogenic induction solution was added to the medium, and the cells were cultured for the next 28 days. The mineralization was assessed by alizarin red staining (Beyotime, Shanghai, China) on the 28th day. After microscopic observation, the coatings dyed with alizarin red were immersed in cetyl chloride (Beyotime, Shanghai, China) for 30 min. Then, the absorbance of the coatings at 562 nm was measured.

#### Alkaline phosphate (ALP) analysis

MC3T3-E1 cells were cultured with different coatings in 24-well plates for 5 days to test ALP activity. First, the phosphatase activity of the culture supernatant was measured using an ALP test kit (Beyotime, Shanghai, China). Meanwhile, the coatings were washed with PBS and fixed with 4% paraformaldehyde for 30 min. Then, the ALP dye solution was added and incubated in the dark for 40 min. Photos were taken by microscope.

#### Wound-healing assay

For the wound-healing assay, 1 mL of HUVECs (5 × 10^4^ cells/mL) was seeded in 24-well plates for 24 h. Images were obtained by optical microscopy, denoted as 0 h. Then, about 1 mm of scratched area was lined using a sterile yellow plastic pipette tip (200 μL) to make a straight wound in the middle of the wells. Next, the medium was replaced by each coated SAL decoction in medium for 5 days. Finally, the culture was incubated for another 24 h to take pictures with a microscope, denoted as 24 h.

#### Cell transwell assay

For the cell transwell assay, 0.5 mL of HUVECs (5 × 10^4^ cells/mL) was seeded in the upper chamber of the transwell plate (Solarbio, Beijing, China) with basic DMEM. Next, 0.5 ml of each coated SAL decoction in medium for 5 days was added to the lower chamber. After 24 h incubation in the cell incubator, the upper chamber was removed, and the cells in the lower chamber were fixed with 5% glutaraldehyde for 20 min. Finally, the cells were stained with crystal violet (Solarbio, Beijing, China) for 30 min and analyzed under a microscope.

#### Matrigel tube formation

To assess Matrigel tube formation, 400 μL/well Matrigel (Solarbio, Beijing, China) was added to 24-well plates and incubated for 30 min. Next, 1 mL of HUVECs (5 × 10^4^/mL) mixed with each coated SAL decoction in medium for 5 days was added to the Matrigel. The cells were incubated for another 6 h. The tube formation was analyzed visually using photographs at 0 h and 6 h, and the tube areas were calculated using ImageJ.

#### Osteogenic and angiogenic gene expression

MC3T3-E1/HUVECs were seeded in a 6-well plate with different coated SAL decoctions in medium for 5 days. After incubation for 48 h, quantitative real-time polymerase chain reaction (qRT-PCR) was performed for MC3T3-E1 and HUVECs. In brief, total RNA was isolated using an RNA extraction kit (Qiagen, Duesseldorf, Germany), complementary DNA (cDNA) was reverse-transcribed using a PrimeScrip RT reagent kit (TaKaRa, Kyoto, Japan) and qRT-PCR was performed with a qRT-PCR detection system (LightCycler 480; Roche, Basel, Switzerland) with SYBRPremix ExTaqII (TaKaRa, Kyoto, Japan). GAPDH served as the house-keeping gene. The procedure was as follows: 95 °C 10 s, 95 °C 3 s, 60 °C 30 s, 72 °C 34 s, and 40 cycles. Genetic expression was calculated using the 2^−△△Ct^ formula. The primer sequences were searched in GenBank sequences and listed in Table 1.

**Table 1:** The PCR primers of osteogenic and angiogenic genes

Genes	Forward primer (5′–3′)	Reverse primer (5′–3′)
Mouse GAPDH	CCCTTAAGAGGGATGCTGCC	ACTGTGCCGTTGAATTTGCC
RUNX2	TTCGCCTCACAAACAACCAC	AACAAAACAAAACGGAGTGAGC
OCN	GAACAGACAAGTCCCACACAGC	TCAGCAGAGTGAGCAGAAAGAT
ALP	GCAGTATGAATTGAATCGGAACAAC	ATGGCCTGGTCCATCTCCAC
Human GAPDH	GAAAGCCTGCCGGTGACTAA	TTCCCGTTCTCAGCCTTGAC
HIF-1α	ACGTTCCTTCGATCAGTTGTCACC	GGCAGTGGTAGTGGTGGCATTAG
MMP-2	CAGGACATTGTCTTTGATGGCATCGC	TGAAGAAGTAGCTATGACCACCGCC
ROCK1	AGCCTACAGATCACTAGCAAT	TGTGGTACTGATGCTCTCCACG
VEGF	ATCGAGTACATCTTCAAGCCAT	GTGAGGTTTGATCCGCATAATC

#### Western blotting assay

For western blotting assay, 2 mL of MC3T3-E1 and HUVECs (5 × 10^4^ cells/mL) were separately seeded in 6-well plates for 24 h. Then, the medium was replaced by each coated SAL decoction in medium for 5 days. Next, the culture was incubated for another 24 h, and protein was extracted using a whole protein extraction kit (Solarbio, Beijing, China). Proteins were quantified with a BCA protein assay kit (Solarbio, Beijing, China). Then, 35 μg protein was separated by 10% SDS–polyacrylamide gel and transferred to a polyvinylidene fluoride membrane (PVDF). After being blocked with 5% non-fat milk for 1 h, PVDF was incubated with different antibodies (RUNX2, OCN, ALP, HIF-1α, MMP-2, ROCK1, and VEGF, Beyotime, Shanghai, China) at 4 °C overnight. Then, the membranes were washed using TBST (0.1% Tween) and detected with secondary antibodies (Beyotime, Shanghai, China). The proteins were detected with HRP substrate by the ChemiDoc™ XRS^+^ System (Yongnuo, Shanghai, China). The level of GAPDH was used as the control.

### In vivo experiments

#### Establishment of animal model of femoral defect

Animal experiments in this study were conducted in accordance with the approval of the Animal Experimentation Ethics Committee of Jiading District Central Hospital Affiliated Shanghai University of Medicine & Health Sciences. Twenty-four 8-week-old male Sprague–Dawley rats (250–300 g) were raised in a specified-pathogen free (SPF) environment. The rats were randomly divided into four groups: (1) Ti, (2) Ti-PDA, (3) 2 mg SAL-PDA and (4) 1 mg DFO (Deferoxamine)-PDA. n = 12. On the 10th day after implantation, 6 rats in each group were killed, and the local inflammatory response was observed by HE staining. On the 30th day after implantation, another 6 rats in each group were killed to observe the local inflammatory response and some special staining. 1 mg DFO-PDA group served as the positive drug control group. Rats were anesthetized with 1.5% sodium pentobarbital (40 mg/kg) via intraperitoneal injection. After the rats were completely anesthetized, the skin was prepared, and the right lower limb of SD rats was disinfected. A longitudinal incision of about 8 mm was made on the right knee joint of SD rats, and the subcutaneous tissue and muscle were separated downward. A hole about 3 mm in diameter and 3 mm in depth was drilled on the lateral femur using a 700^#^ split drill. The Ti uncoated and coated implants (Φ = 3 mm, h = 3 mm) were slowly placed into the defect area until all of them entered the bone. Then the muscle and skin were sutured layer by layer to seal the wound. The judging index of successful model was the bone defect area detected by X-ray.

#### New bone formation analysis

On the 10th, 20th and 30th day after implantation, X-ray images of the implanted area of the femur defect were taken using a small animal X-ray imaging system (X5600, SEAMARK, Suzhou, China) to evaluate the healing of the Ti implant and the surrounding bone tissue. At the same time, the rats were anesthetized and euthanized on the 30th day after implantation, and the femoral samples containing Ti implants were scanned by micro CT (SkyScan 1272, DKSH, Shanghai, China) to evaluate the new bone formation on the surface of Ti implants in different groups. The MIMICS medical image control system and 3-matic engineering software were used for 3D reconstruction. 3D reconstruction was used to quantitatively analyze the images to determine the bone mineral content (BMC), bone mineral density (BMD), bone volume density (BV/TV), trabecular thickness (Tb.Th), bone area density (BS/TV) and trabecular number (Tb.N).

#### HE staining and IHC analysis

The right femur specimen was collected on the 30th day after surgery. The sample was then fixed with formalin for 24 h. After fixation, the sample was decalcified with a 10% EDTA decalcified solution (pH = 7.2) (Solarbio, Beijing, China). Briefly, the sample was decalcified at 37 °C for 28 days, and the decalcified solution was renewed every 3 days. After the decalcification was completed, the sample was embedded in the paraffin wax, and the coating implanted area was cut into continuous sections about 3 μm thick with a microslicer. Sections were stained with HE to assess osteogenesis progression at 30 days postoperatively. In addition, to evaluate the osteogenic activity and angiogenesis, the expression of COL-1 and CD31 in the coating implantation area was measured by IHC using COL-1 and CD31 antibodies (Beyotime, Shanghai, China) and analyzed by ImageJ software.

#### Goldner's trichrome analysis

The Goldner's trichrome was purchased from Solarbio, Beijing, China. Firstly, the slices were dewaxed with xylene, rinsed with absolute ethanol and fully rinsed with distilled water. The prepared Weigert iron hematoxylin was added and dyed for 15–20 min. After washing with running water for 1 min, Acid Ponceau staining solution was added for 5 min. The slices were washed with weak acid working solution for 15–30 s. Orange G staining solution for staining was added until Ponceau staining solution was removed, which generally took 3–10 min. The slices were rinsed with the prepared weak acid working solution for 15–30 s. The slices were added into bright green staining solution for dyeing for 5 min and rinsed with the prepared weak acid working solution for 3 times, 15 s each time. Finally, the slices were rinsed with distilled water, sucked or air dried, dehydrated with absolute ethanol, sealed with neutral gum, and observed under microscope.

#### Safranin O-fast green staining

The specimens were routinely fixed, decalcified, paraffin embedded and sectioned, and then routinely dewaxed to water. Then, the slices were stained with freshly prepared Weigert dye for 3–5 min and washed with water. The specimens were soaked in solid green staining solution for 5 min and quickly washed with weak acid solution for 10–15 s to remove the residual solid green. The specimens were added into Safranin O-fast green (Solarbio, Beijing, China) and soaked for 5 min. Dehydrated with 95% ethanol and absoluted ethanol respectively. Xylene was transparent and sealed with optical resin. Finally, the specimens were observed under microscope.

### Detenination of contents of TNF-α and IL-6 in serum

After coatings were implanted into the rats model of femur defect for 7 days, 2 mL blood was collected from tail vein. The whole blood was placed at 37 °C for 0.5 h, centrifuged at 3500–4000 rpm for 5–10 min, and the serum was separated. The secretion levels of TNF-α and IL-6 in serum were determined according to the operation instructions of rat ELISA kit (Abcam, Cambridge, UK), and the corresponding contents were calculated.

### Statistical analysis

There were three replicates for each cell experiment and no less than three samples for each animal experiment. The results were expressed as mean ± mean standard error (SEM). Statistical analysis was performed using GraphPad Prism 5.0 (San Diego, CA, USA) software. Two-way analysis of variance was used for all data. The significance level was set at 0.05, and P < 0.05 was considered statistically significant.

## Results

### Characterization of the SAL-loaded coatings

**Fig. 1:**
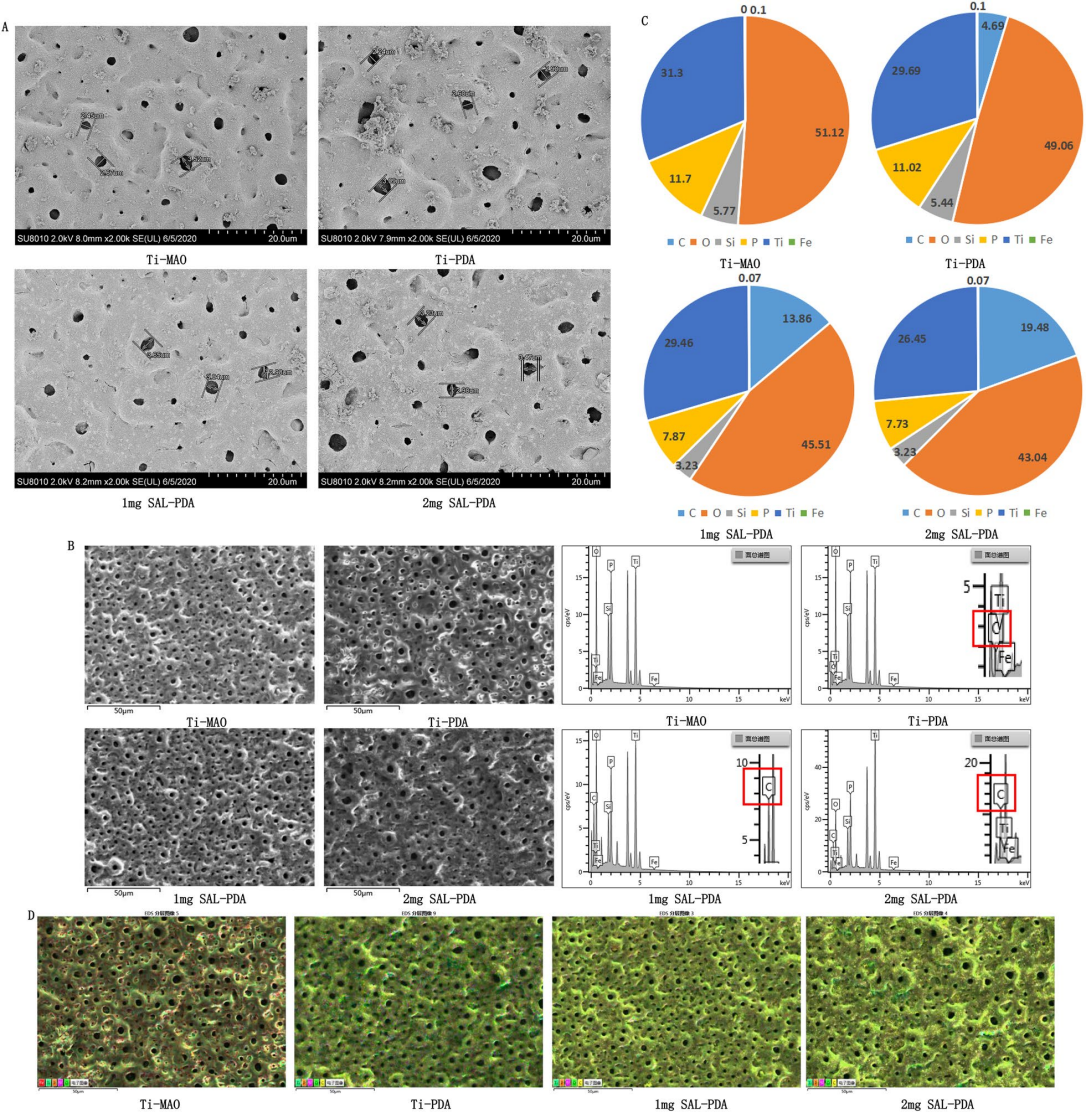
Characterization of materials. **A** The surface morphology of different coatings by SEM. **B** Surface element contents of different coatings by EDS. The solid red box showed the percentage of carbon. **C** Mass percentage (Wt%) of element content in different coatings. The total percentage was 100%. **D** The distribution of element C on the surface of the coating was analysed by EDS mapping. The more carbon, the darker the yellow. Scale bar = 50 μm

SEM analysis showed that after MAO treatment, a microporous structure was successfully constructed on the surface of pure Ti. The average pore sizes of the four groups (Ti-MAO, Ti-PDA, 1 mg SAL-PDA and 2 mg SAL-PDA) were 2.88 μm, 2.71 μm, 3.02 μm and 3.23 μm, respectively. There was no significant difference between the groups. The surface morphology was shown in Fig. 1A. The results showed that the surface modification of MAO by PDA had no effect on the pore morphology, and the coating still had good microporous characteristics.

EDS analysis showed that active elements Si and P were successfully incorporated into pure Ti after MAO treatment (Fig. 1B). Compared with the Ti-MAO group, the distribution of C in the Ti-PDA group was found, which indicated that PDA was successfully loaded into the surface of MAO. With the increase of SAL concentration, the content of C on the coating surface also increased, which indirectly indicated that SAL was successfully loaded on the coating surface. Figure 1C showed the mass percentage of element content of each coating. According to the mapping results of the energy spectrometer, element C was distributed evenly on the coating surface, as shown in Fig. 1D. With the loading of PDA and SAL, the content of element C on the surface of the coating increaseed, and the darker the maping color was, which indirectly indicated that the PDA and SAL were successfully loaded on the surface of the coating.

The loading amount of PDA on the coating was 29.67 ± 2.12 μg/mm^3^, the drug loading of 1 mg/mL SAL on the coating was 11.89 ± 1.87 μg/mm^3^, and 2 mg/mL SAL on the coating was 15.53 ± 0.79 μg/mm^3^. These characterization tests indicated that both PDA and SAL were successfully loaded on the titanium micro-arc oxidation coating.

### Release rate of SAL

**Fig. 2:**
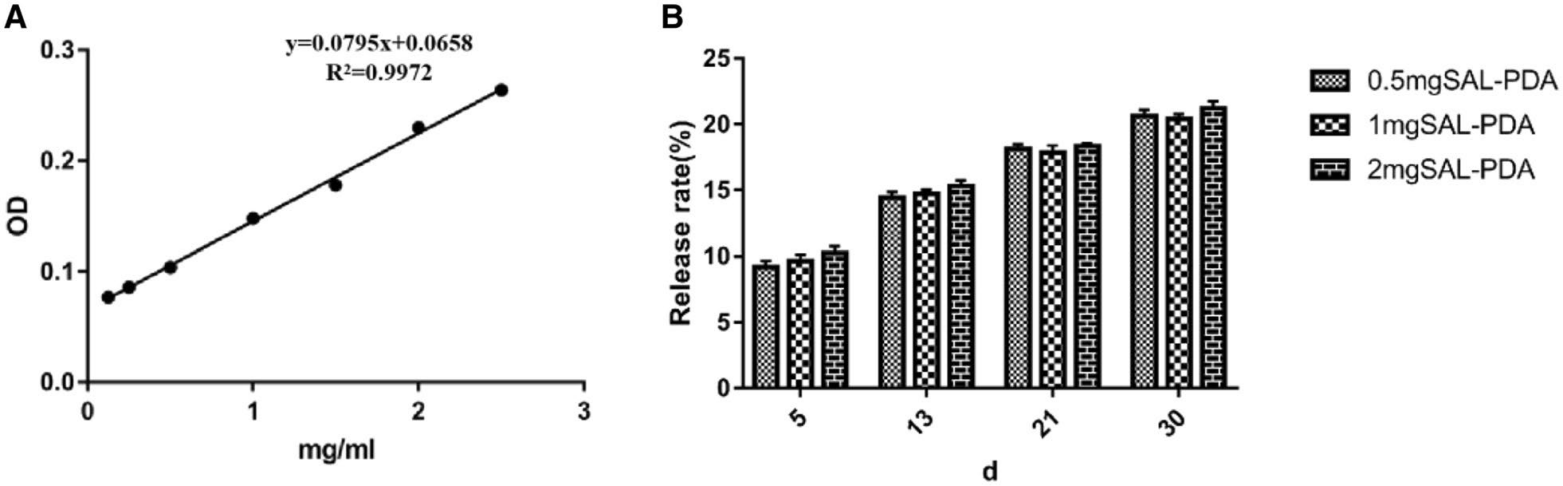
Release rule of SAL. **A** Standard curve of drug concentration absorbance. **B** Cumulative release percentage of SAL on different days

As shown in Fig. 2, the cumulative release rate of SAL loaded with PDA on Ti surface was relatively flat and slow across days 5–30. The above data suggested that SAL loaded on Ti coating could achieve slow and stable release without sudden release risk. On the 5th day, the SAL release rates of the 1 mg SAL-PDA group and 2 mg SAL-PDA group were 9.69 ± 0.77% and 10.19 ± 0.85%, respectively.

### SAL-incorporation enhanced osteogenesis in vitro

**Fig. 3:**
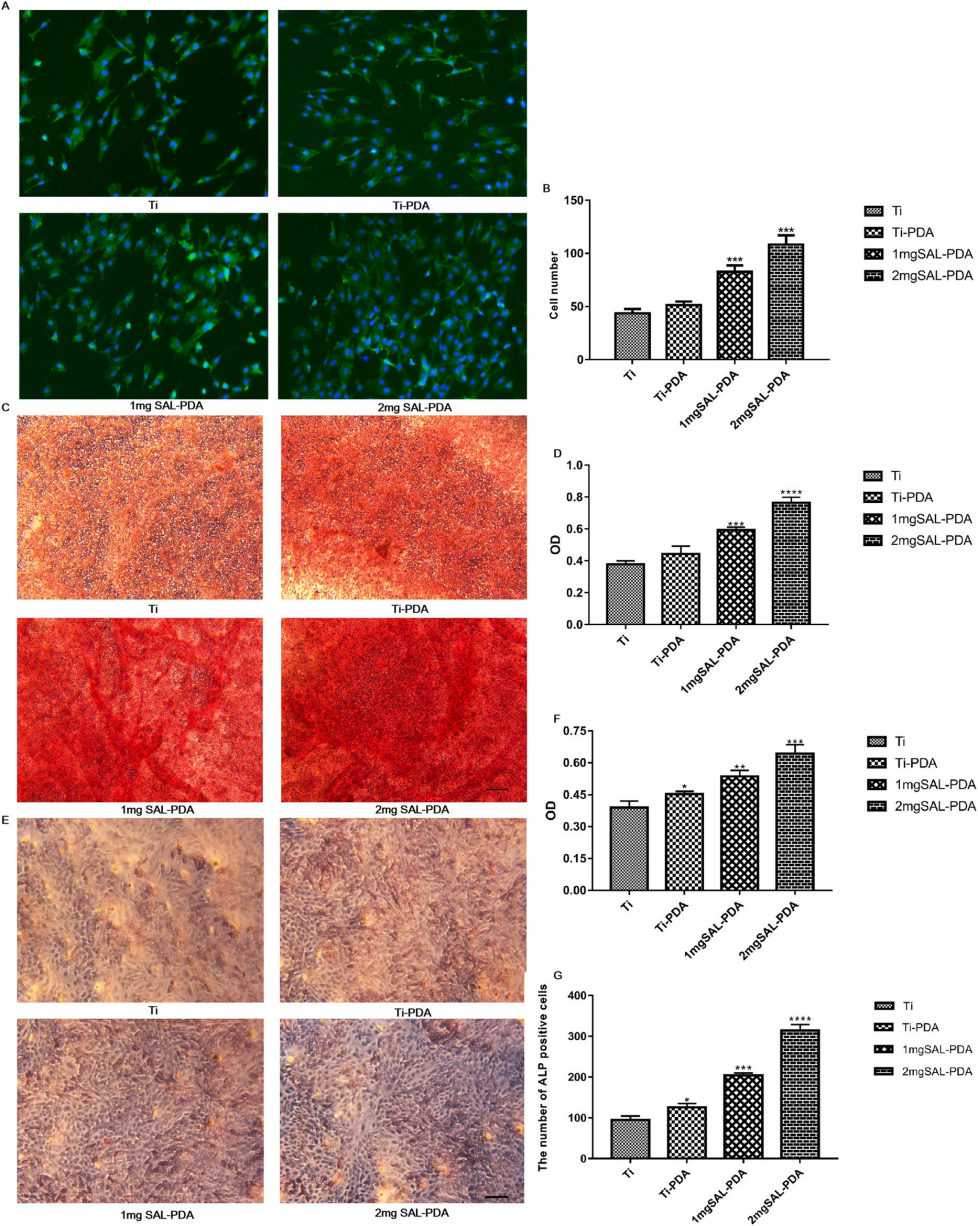
Osteogenesis of MC3T3-E1 cells cultured in different coatings in vitro*.* **A** Phalloidin and DAPI staining on the 5th day. Microscope magnification was ×200. Scale bar = 100 μm. **B** Numbers of stained cells. **C** Alizarin Red staining on the 28th day. **D** Quantitative results of Alizarin Red staining. **E** ALP staining of MC3T3-E1 cells on the 5th day. **F** ALP content in supernatant of each coating group. **G** The number of ALP positive cells. Microscope magnification was ×100. Scale bar = 200 μm. *P < 0.05, **P < 0.01, ***P < 0.001 and ****P < 0.0001 were obtained by comparison with uncoated Ti

The number and morphology of cells were observed under microscope. Compared with the control groups, the number of cells in the 2 mg SAL-PDA group was higher. At the same time, the cell morphology in the 2 mg SAL-PDA group was better, the cell spreading area was larger, the cell growth condition was better, the cell granule was more complete and the connection with adjacent cells was better (Fig. 3A, B).

The results of cell calcification staining showed that there was a certain degree of staining in each group, but the staining in the 2 mg SAL-PDA group was more obvious, suggesting greater promotion of bone differentiation (Fig. 3C). Then, each group were dissolved with 10% cetylpyridinium chloride, and the absorbance was measured at a wavelength of 562 nm. It was shown that the 2 mg SAL-PDA group had a significantly increased ability to promote cell mineralization (Fig. 3D). Therefore, by loading SAL on the surface of the TiO_2_/SiP coating, the ability of the coating to promote cell mineralization could be further improved.

ALP staining results showed that the staining phenomenon was more obvious in the 2 mg SAL-PDA group, and the number of ALP positive cells was greater (Fig. 3E, G). At the same time, ALP detection of the cell supernatant of each group showed that the ALP content of the 2 mg SAL-PDA group was higher (Fig. 3F). The results showed that SAL could promote bone formation by promoting ALP release.

### SAL-incorporation enhanced angiogenesis in vitro

**Fig. 4:**
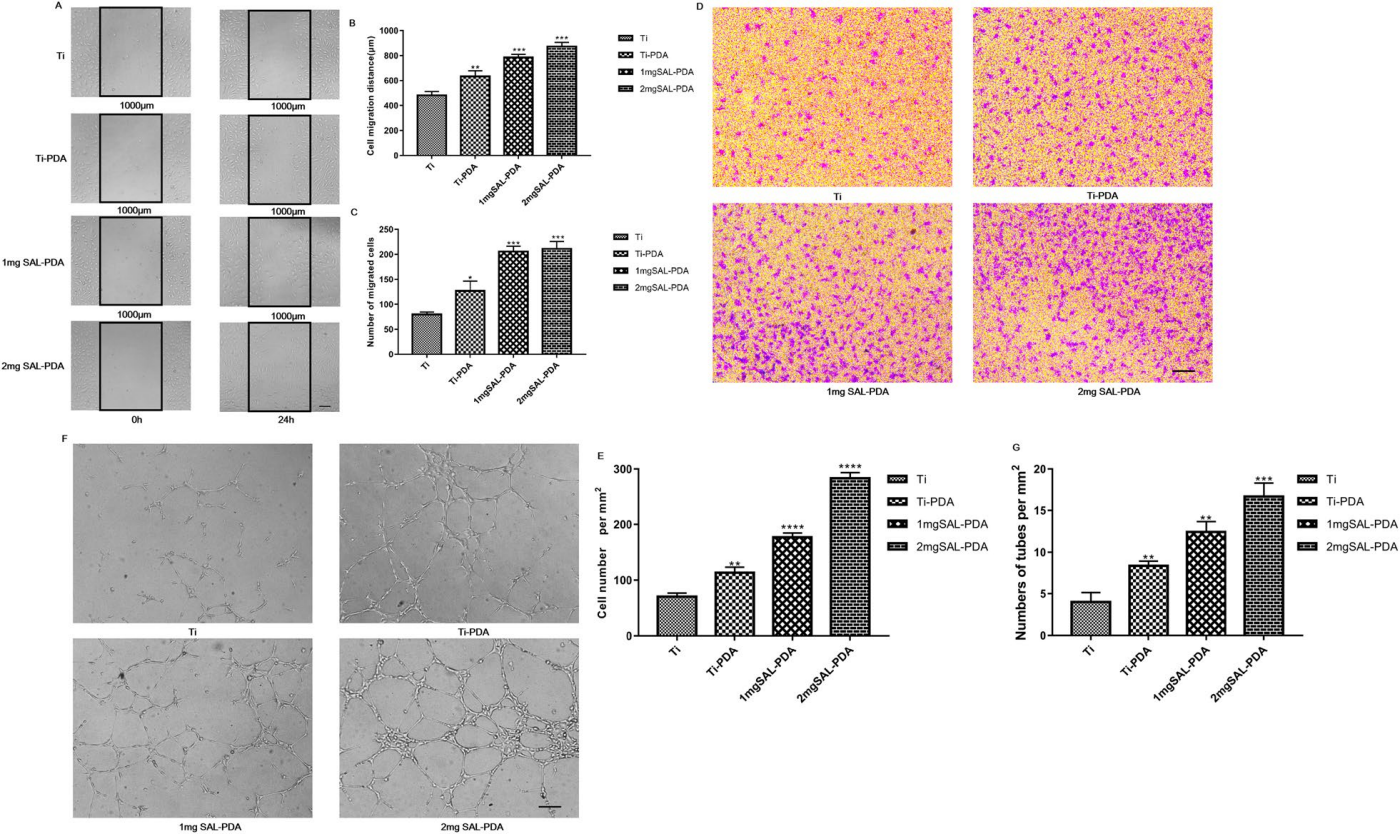
Angiogenesis of HUVECs cultured in different coatings in vitro*.* **A** Maps of wound healing scratch. **B** Migration distance of HUVECs. **C** The number of migrated cells. **D** Images of the transwell experiment. **E** Number of cells migrating to the inferior chamber. **F** Tube formation in vitro. **G** Statistical analysis of tube number. Microscope magnification was ×100. Scale bar = 200 μm. *P < 0.05, **P < 0.01, ***P < 0.001 and ****P < 0.0001 were obtained by comparison with uncoated Ti

The results of wound healing scratch assay are shown in Fig. 4A–C. SAL-PDA promoted the migration of HUVECs. The distance of migration and migration index of 2 mg SAL-PDA were larger compared with other groups. Figure 4D, E shows the results of transwell assay. The 1 mg SAL-PDA and 2 mg SAL-PDA groups had more cell migration to the lower compartment of the transwells compared with the control group. The results showed that SAL coatings could promote the migration of endothelial cells.

Tube formation experiments were conducted to test the angiogenic properties of coatings. Complete tube formation was observed in the four groups. However, SAL-PDA induced more tubes and connected nodules than other groups (Fig. 4F, G). The result was consistent with migration assays. The 2 mg SAL-PDA group showed a better ability to form tubes over other coatings. The experiments indicated that SAL coatings had stronger pro-angiogenic properties.

### Detection of genes and proteins related to osteogenesis and angiogenesis

**Fig. 5:**
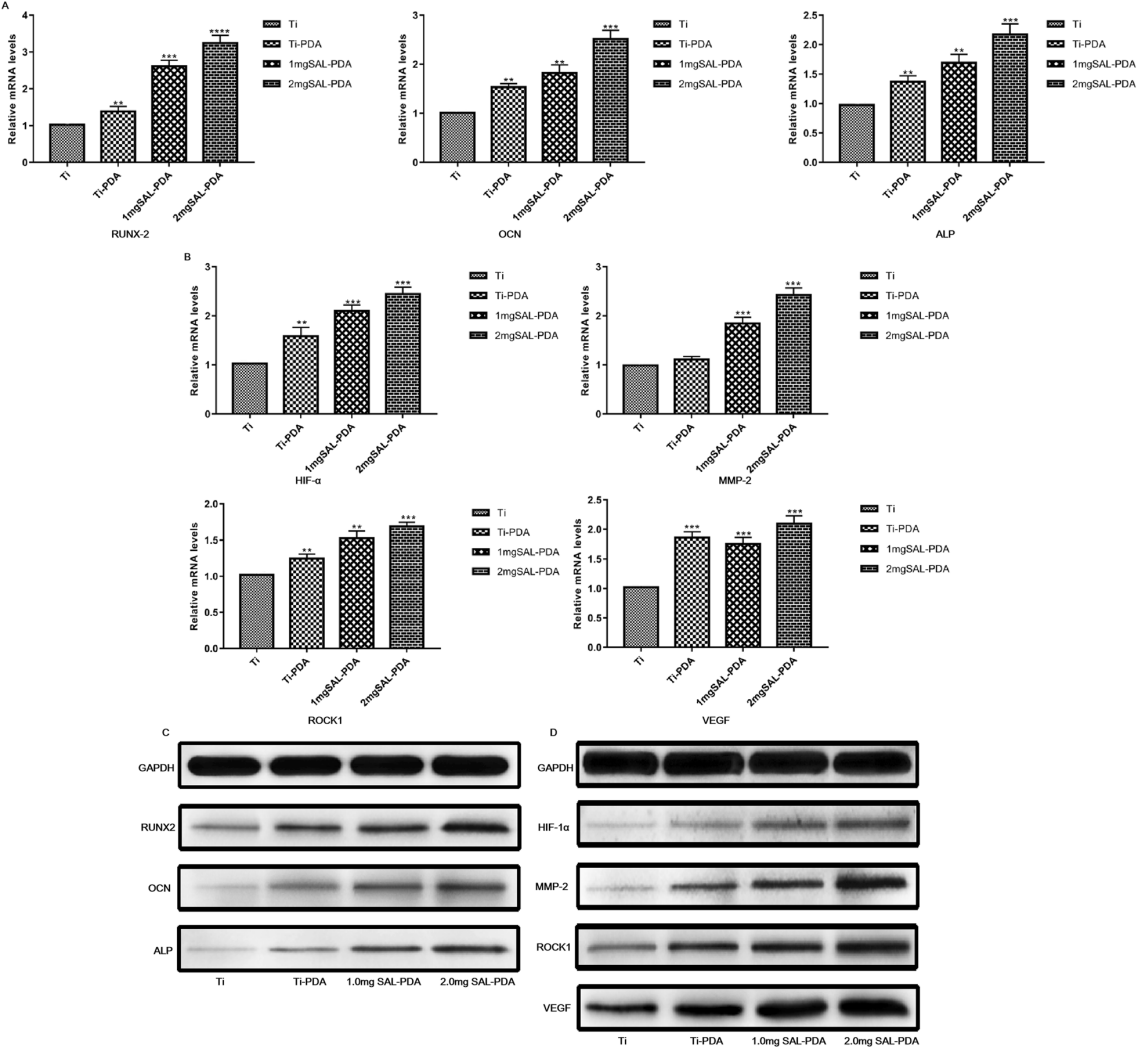
Genes and proteins related to osteogenesis and angiogenesis. **A** Fold increases of RUNX2, OCN and ALP genes. **B** Fold increases of HIF-1α, MMP-2, ROCK1 and VEGF genes. **C** Western blot of RUNX2, OCN and ALP proteins. **D** Western blot of HIF-1α, MMP-2, ROCK1 and VEGF proteins. All values were normalized to GAPDH. **P < 0.01, ***P < 0.001 and ****P < 0.0001 were obtained by comparison with uncoated Ti

We conducted genetic level (PCR) and protein level (western blot) tests to assess the osteogenic and angiogenic properties of SAL coatings in vitro. RUNX2 is an essential transcription factor for osteoblast differentiation and bone formation. OCN is a gene that regulates osteoblastogenesis. ALP is a marker of early osteogenesis. HIF-1α induces the expression of its downstream target genes, especially VEGF, and participates in bone and vascular coupling. MMP-2 can promote angiogenesis during osteogenesis. ROCK1 can promote endothelial cell proliferation, migration, adhesion and angiogenesis. VEGF is a regulatory factor in the process of bone angiogenesis. In conclusion, by detecting the content of related genes and proteins, the possible targets of the SAL coating in promoting osteogenesis and angiogenesis were analyzed. As shown in Fig. 5A, B, the expression of genes related to osteogenesis and angiogenesis in SAL coating groups was significantly increased. At the same time, the expression levels of the corresponding proteins regulated by the enhanced genes were also increased (Fig. 5C, D). The expression of genes and proteins was higher in the 2 mg SAL-PDA group.

### SAL incorporation enhanced osteogenesis and angiogenesis in vivo

#### X-ray examination results of the implanted area

**Fig. 6:**
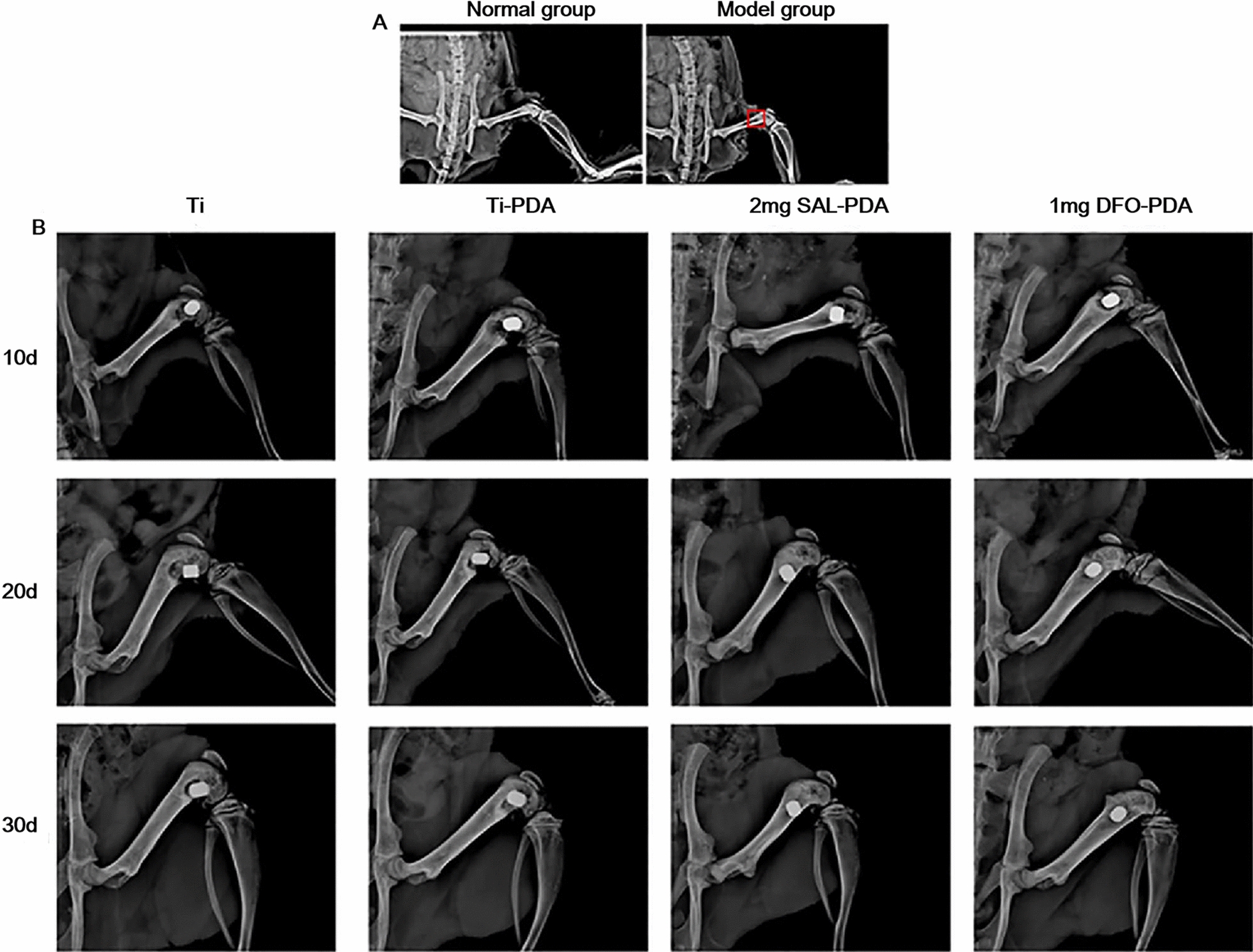
X-ray results of each coating group. **A** X-ray of femur of normal rats and model rats. The red box indicates the bone defect area. **B** X-ray images of the implanted area of femoral defect in each group after operation

An X-ray image of a bone defect after modeling was shown in Fig. 6A. X-ray examination was performed on the 10th, 20th and 30th day after operation to observe the bone healing between Ti implants and surrounding defects (Fig. 6B). On the 10th day after operation, the standard ortho X-ray films showed that the Ti materials in each group had good implantation position, normal adhesion to the surrounding bone tissue and no loosening or obvious falling off. There were many voids around the titanium materials in each group. On the 20th day after operation, the bone defect areas of the Ti and Ti-PDA groups showed some of the voids and some low-density radiography. In the SAL-PDA and DFO-PDA groups, the surrounding space was basically healed, and there were many low-density angiographic areas around. Compared with the control group, the healing was better. On the 30th day after operation, compared with the control group, the defect area around the material in the SAL-PDA and DFO-PDA groups healed better. The material combined better with the surrounding bone tissue, and the boundary around the defect was better blurred. There was no bone resorption or dissolution, and the replacement of the surrounding defect area by new bone tissue was more obvious.

#### Analysis of new bone formation on each coating surface

**Fig. 7:**
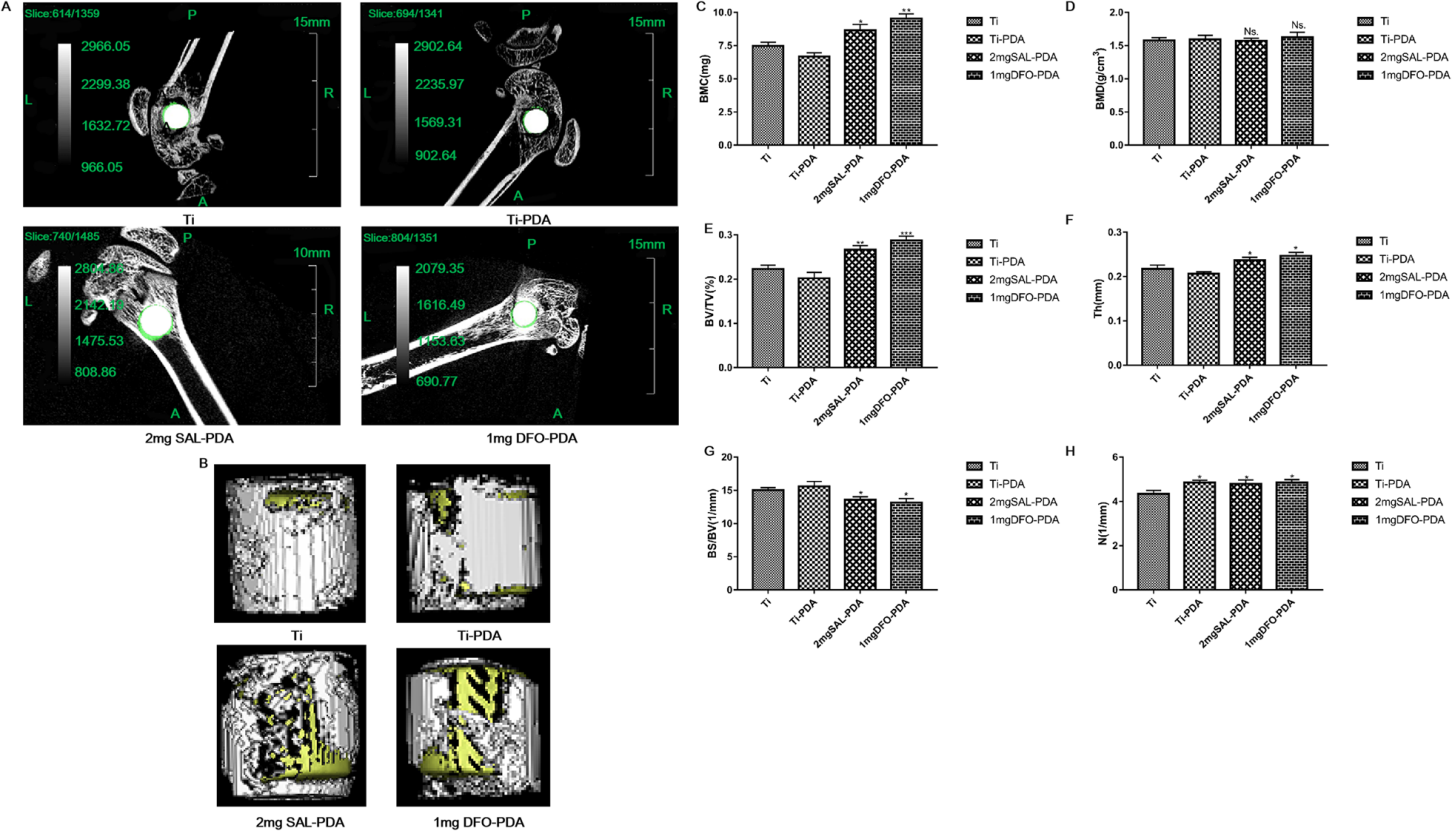
Analysis of new bone formation on Ti implant surface. **A** The maps and analysis of micro CT scanning. **B** 3D reconstruction of micro CT. The white part was titanium, and the yellow part was the new bone tissue around titanium. **C** Analysis results of BMC. **D** Analysis results of BMD. **E** Analysis results of BV/TV. **F** Analysis results of Th. **G** Analysis results of BS/BV. **H** Analysis results of N. *P < 0.05, **P < 0.01 and ***P < 0.001 were obtained by comparison with uncoated Ti

On the 30th day after operation, the femur samples of rats with Ti implants were collected and analyzed by micro CT scanning and 3D reconstruction. Figure 7A was a micro CT diagram. Figure 7B was a 3D reconstruction of micro CT. It showed that SAL coating promoted new bone formation. We found that the contents of BMC, BV/TV, Th and N in the 2 mg SAL-PDA group were higher than those in the Ti group. BMD is an important indicator of bone quality, and low density will lead to osteoporosis. The higher the BMD, the harder and more brittle the bones are. The smaller BS/BV is, the closer the shape of the bone is to the ball, the more uniform the stress is and the more pressure it can bear (Fig. 7C–H). The results suggested that loading SAL on the surface of Ti implants could improve the osseointegration of Ti implants. The results were consistent with the qualitative results of X-ray examination.

#### Analysis results of HE and IHC staining

**Fig. 8:**
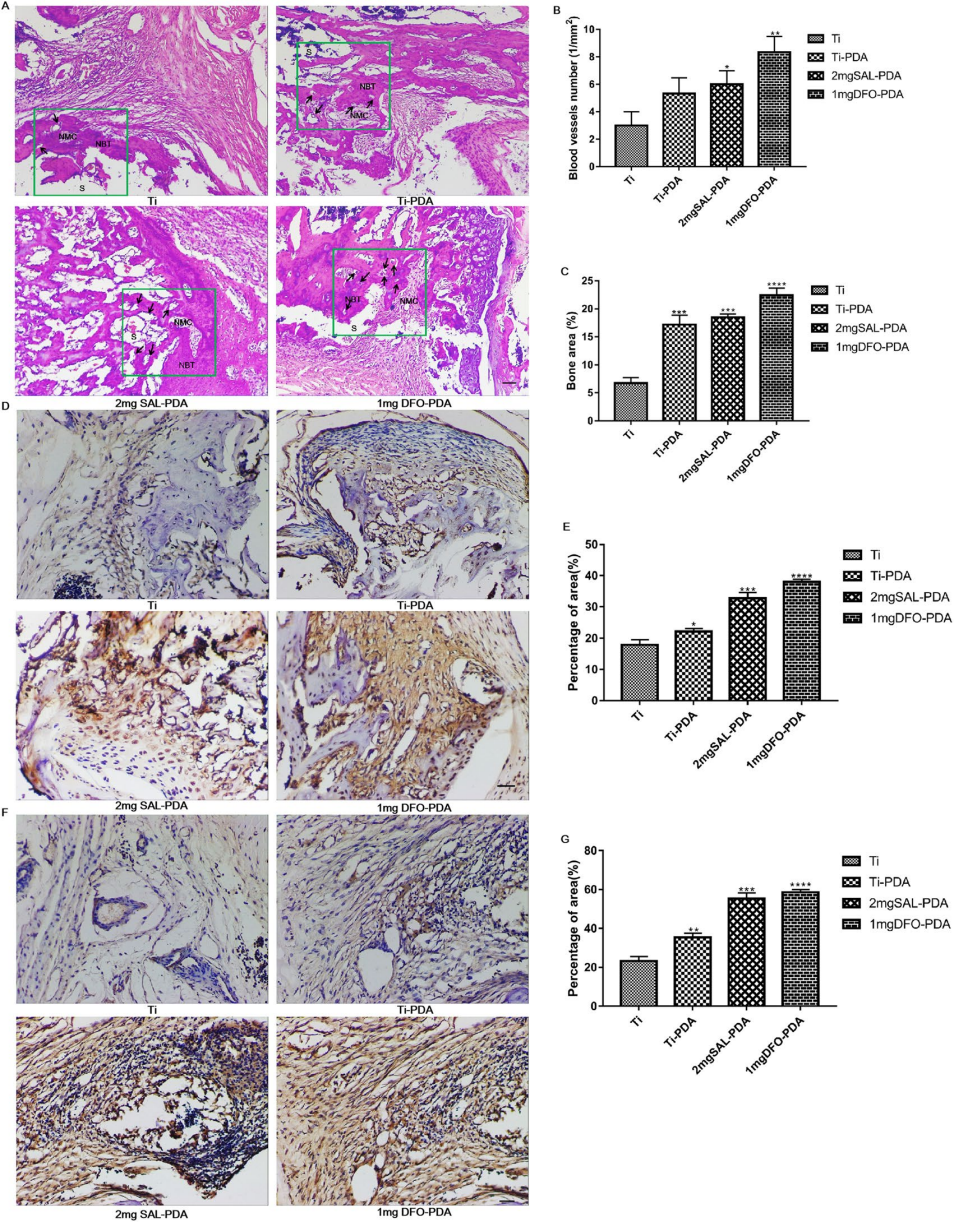
Histological analysis of osteogenesis and angiogenesis after coatings implantation. **A** HE staining during bone formation. S denotes the coating implantation site, NBT denotes the new bone trabecula, NMC denotes the new bone marrow cavity and the black arrow indicates the new blood vessels. **B** The numbers of neovascularization. **C** The areas of neovascularization. **D** The expression of COL-1 on the 30th day after coatings implantation. **E** The percentage of area of COL-1 signals. **F** The expression of CD31 on the 30th day after coatings implantation. **G** The percentage of area of CD31 signals. Microscope magnification was ×200. Scale bar = 100 μm. *P < 0.05, **P < 0.01, ***P < 0.001 and ****P < 0.0001 were obtained by comparison with uncoated Ti

According to the results of HE staining (Fig. 8A), all groups showed osteogenesis in the coating-implanted region on the 30th day after surgery. After implantation, the proliferation of newly formed callus (woven bones) was more obvious in the 2 mg SAL-PDA and 1 mg DFO-PDA groups, which correlated with the micro-CT analysis of higher BV/TV and N parameters compared to uncoated Ti. In addition, the callus in the coating implant site of rat femur was replaced by dense lamellar bone, which was composed of trabecular bone and cancellous bone, and the newly formed trabecular bone and marrow cavity were distributed regularly. In addition, the trabecular morphology and density of the 2 mg SAL-PDA group were better compared with uncoated Ti, which indicated that the SAL coating could synergistically promote bone formation. At the same time, compared with the control group, the numbers of new blood vessels and the area of new bone in the SAL coating group were greater (Fig. 8B, C).

After 30 days of implantation of coatings into the rat bone defect model, the peripheral areas of bone defects were stained with COL-1 IHC. As shown in Fig. 8D, E, the low expression of COL-1 was observed in the Ti and Ti-PDA groups, and the expression of COL-1 was significantly higher in the 2 mg SAL-PDA and 1 mg DFO-PDA groups. The results suggested that new bone formation on the Ti surface was promoted by SAL loading on the surface. IHC staining of CD31 was performed in the area around the bone defect, as shown in Fig. 8F, G. Less CD31 expression was observed in the Ti and Ti-PDA groups, and CD31 was significantly higher in the 2 mg SAL-PDA and 1 mg DFO-PDA groups. Quantitative analysis showed that SAL could collectively promote the angiogenesis of the Ti surface.

#### Analysis of Goldner's trichrome and Safranin O-fast green staining

**Fig. 9:**
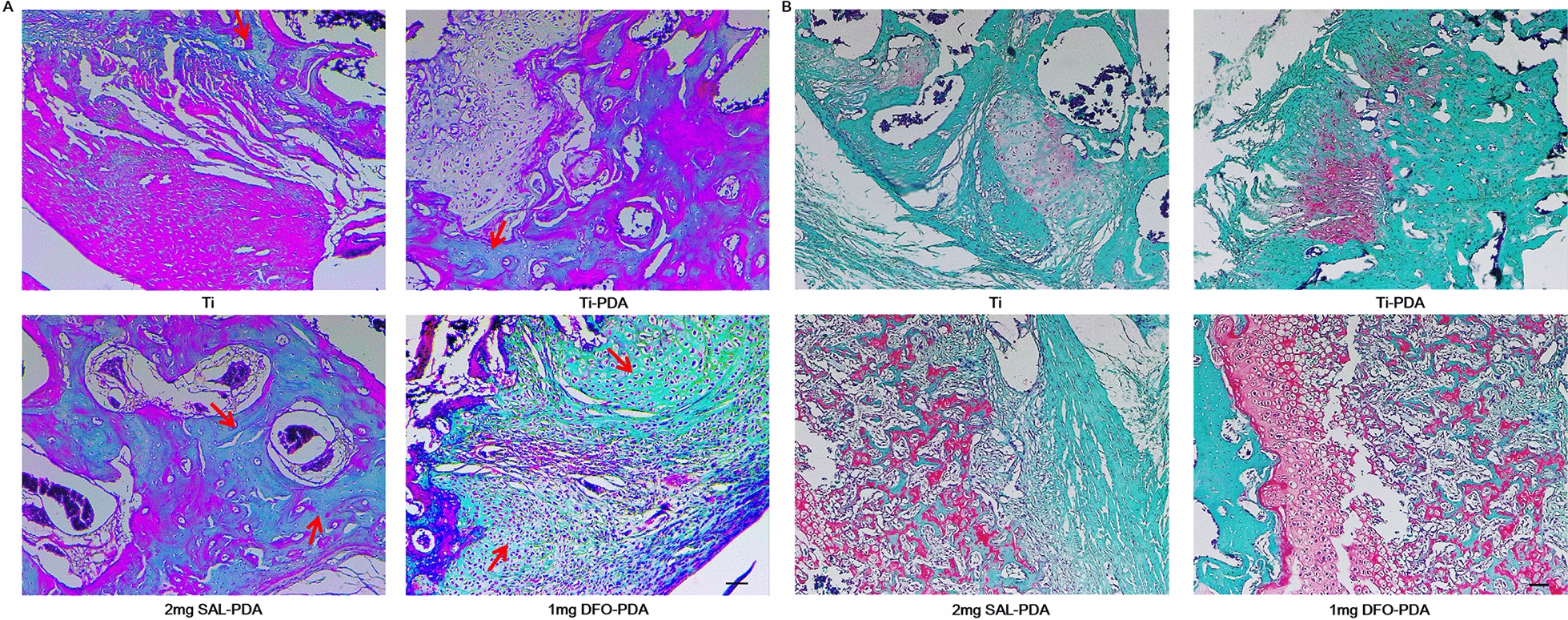
Histological special staining. **A** Goldner trichrome staining. Mineralized bone was dyed green. Red arrows represented mineralized bone formed. **B** Safranine O-fast green staining. The cytoplasm, muscle, collagen fibers and bone tissue were stained gray green, the cartilage nucleus was dyed blue, and the chondrocyte cytoplasm was dyed red. Microscope magnification was ×100. Scale bar = 200 μm

In order to explore the maturity of new bone tissue around the implantation site in different experimental groups, the tissue sections of animal experimental samples were stained with Goldner trichrome. The experimental results were shown in Fig. 9A. The results showed that a small amount of mineralized bone appeared near the implantation site in Ti and Ti-PDA groups. Mineralized bone formation around the implant site in the 2 mg SAL-PDA and 1 mg DFO-PDA groups was significantly increased. At the same time, the newly mineralized bone in 2 mg SAL-PDA and 1 mg DFO-PDA groups was denser and the thickness of mineralized bone was higher. It was shown that SAL coating could effectively promote the formation of new bone and was expected to shorten the time required for bone defect healing.

At the same time, modified Safranine O-fast green staining was carried out. The results of modified Safranine O-fast green staining showed that the defect areas in Ti and Ti-PDA groups were mainly gray-green muscles and collagen fibers, with poor osteogenesis. More new bone formation, more cartilage tissue and uniform quality were observed in 2 mg SAL-PDA and 1 mg DFO-PDA groups, and the cortical bone connection was in the plastic stage (Fig. 9B). The experimental results showed that SAL coating could promote osteogenic activity.

#### Analysis of inflammation in rats after coating implantation

On the 1st to 7th day of the experiment, the observation showed that the feeding, activity, feces and other general conditions of the coated rats were normal, and their hair was bright. There was slight local redness and swelling at the implantation site of the coating, but there was no temperature increase or purulent secretions on the skin surface adjacent to the wound. The body weight of rats increased gradually. These findings suggested that the implant produced only a mild inflammatory response.

**Fig. 10:**
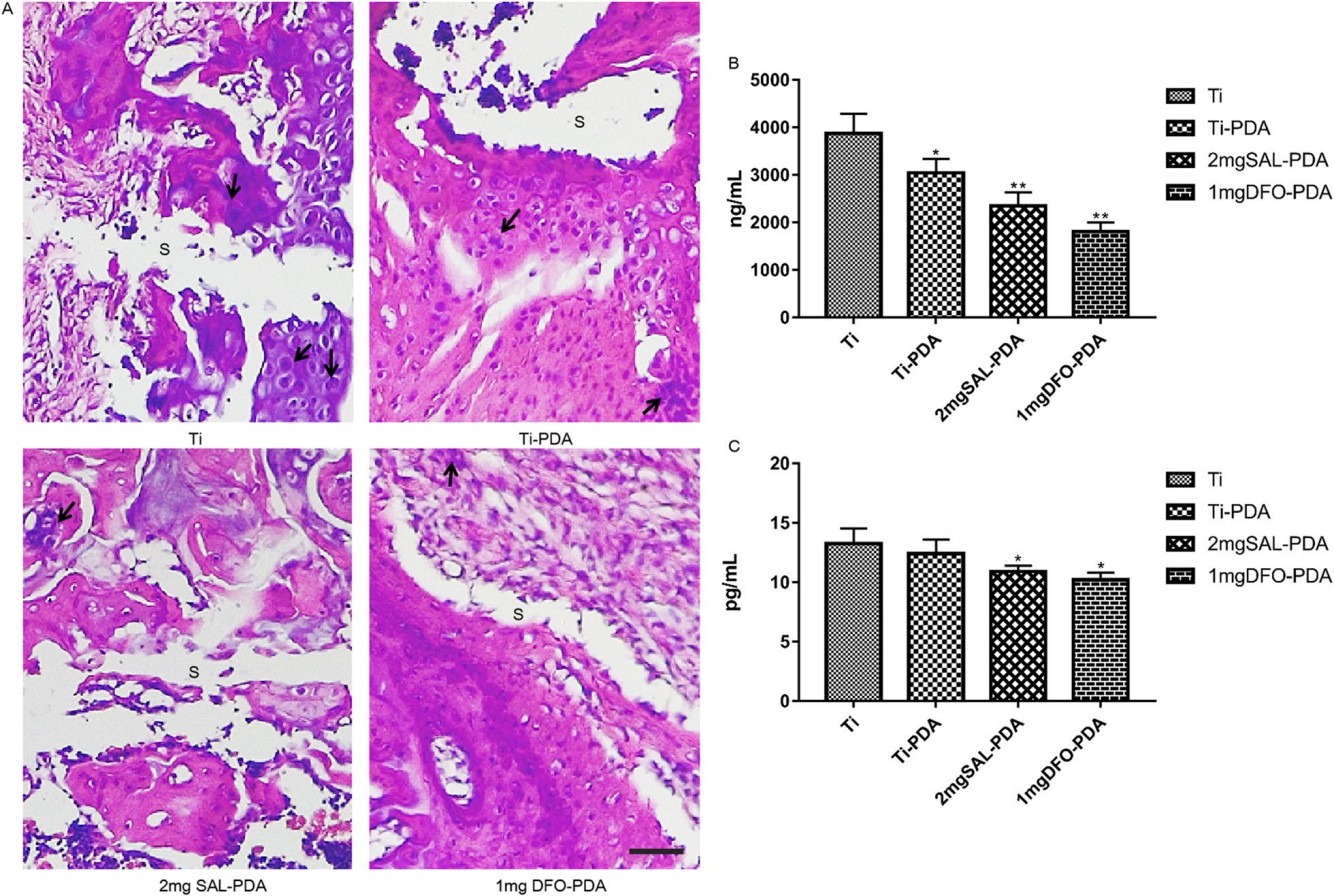
Analysis of inflammation in rats with coatings. **A** HE staining during bone formation on the 10th. S denotes the coating implantation site, and the black arrow indicates the macrophages. Microscope magnification was ×400. Scale bar = 25 μm. **B** Content of TNF-α in serum. **C** Content of IL-6 in serum. *P < 0.05 and **P < 0.01 were obtained by comparison with uncoated Ti

HE staining on the 10th day of implantation showed that the implant adhered closely to the surrounding tissues. It could be seen that there were tissues growing around the implant, and there were manifestations of acute inflammation in the tissues, mainly macrophages. However, compared with the control group, the number of inflammatory cells in SAL coating group was less and the inflammatory reaction was mild (Fig. 10A). HE staining on the 30th day of implantation showed that fibrous tissue hyperplasia was more obvious in the tissues growing around the implant, there were more neovascularization in the tissues, but the inflammatory cells were less than before, indicating that the early inflammatory reaction was heavier and decreased with time (Fig. 8A).

To further investigate whether SAL had anti-inflammatory effect, we detected the secretion levels of TNF-α and IL-6 in serum of rats in each group. TNF-α is an important inflammatory factor. It has been found that TNF-α is directly involved in the pathophysiological process of inflammation. TNF-α is one of the strongest mediators of inflammation in the body. It can induce, coordinate and regulate other inflammatory factors such as IL-6 in the process of inflammatory response [15]. The secretion levels of TNF-α and IL-6 were detected by ELISA. The results showed (Fig. 10B, C) that compared with Ti control group, serum TNF-α and IL-6 levels in 2 mg SAL-PDA and 1 mg DFO-PDA groups were significantly decreased. The results showed that SAL coating could partially inhibit the inflammatory response of femoral defect and implanted medical materials in rats. It could reduce the secretion of cytokine TNF-α to regulate the immunity of rats.

## Discussion

Medical implants are very important for clinical bone defect repair. In the past 5 years, the demand for implants has increased dramatically. For example, the number of hip revision surgeries has increased by 26%, and it is expected to reach 137% by 2030 [16]. Ti and Ti alloy have been widely used in the field of orthopedic implants due to their excellent mechanical properties and structural stability [17]. However, the surface biological activity of Ti implants is low, and it only forms a simple mechanical interlock with bone tissue, without osteointegration performance, so it is prone to poor fixation after implantation. In addition, the surface of Ti implants lacks effective antibacterial activity, which makes the implant prone to bacterial infection, leading to postoperative infection complications [18, 19]. To overcome the problems caused by the surface defects of Ti implants, various surface modifications of Ti implants can be conducted to improve the osseointegration and antibacterial properties of the surface of Ti implants. Surface modification can promote early bone healing and reduce the risk of implant infection.

Surface modification methods of Ti implants mainly include mechanical methods, physical methods, chemical methods and biochemical methods [20]. Chemical methods include chemical treatment (pickling, alkali treatment and passivation) and electrochemical treatment (anodic oxidation, MAO and electrodeposition). Chemical methods can be used to prepare various microstructure and nanostructure coatings on the surface of Ti or to incorporate a variety of biological active elements. The biochemical method introduces active substances, such as proteins, peptides, enzymes and small molecule drugs, to the surface of the Ti implant so as to enhance the biological activity of the surface of the Ti implant and accelerate the integration ability of the implant with the surrounding bone tissue. MAO technology has been proved to be an effective surface treatment method for enhancing the osseointegration of Ti implants, and porous coatings doped with a variety of bioactive elements have been developed [21]. In our research, a porous and micro-structured TiO_2_/SIP coating was constructed on the surface of Ti implants by MAO, and SAL was loaded on the surface of the coating by subsequent biochemical modification technology to study the bone integration performance of the composite porous Ti implant. Dopamine contains a catecholamine functional group and a lysine terminal amino group, which can self-assemble into a strong adhesive PDA nanolayer on the surface of glass, metal, ceramic, organic matter and other materials in an alkaline environment. PDA bound to the surface of the material has the ability to adsorb growth factors and small molecular compounds [22]. Therefore, in this study, SAL was loaded onto the Ti coating with PDA.

Traditional Chinese medicine has accumulated a great deal of clinical experience in promoting bone healing, and many active compounds of traditional Chinese medicine have been found to have good bone promoting activity [23, 24]. SAL, the main active compound extracted from the roots of Rhodiola, has a variety of pharmacological effects [25]. Mei found that SAL promoted the proliferation and osteogenic differentiation of human osteoblast-like cell lines by regulating the cell cycle distribution and upregulating the expression of RUNX2 and Osterix [26]. The latest study reported that SAL could significantly promote the proliferation, differentiation and mineralization of rat calvarial osteoblasts [27]. PDA cannot change the biological inertia of the Ti surface due to the lack of direct osteogenic activity of its modified Ti implants. Therefore, SAL with osteogenic activity was loaded onto the surface of Ti with PDA carrier to study its effect on the osteogenic ability of Ti surface in this study.

Bone is a highly vascularized tissue that depends on the close relationship between blood vessels and bone cells to maintain bone integrity. Therefore, angiogenesis plays a key role in bone development, repair and remodeling. Previous studies have shown that angiogenesis and bone formation are coupled, involving a variety of cytokines, signaling pathways and microRNA [28]. BMP-Smads, Wnt/β-Catenin, MAPK, PI3K/AKT, estrogen and other signaling pathways mainly promote bone formation by acting on osteoblasts. The gene and protein expressions of RUNX2, OCN, ALP and COL-1 in these signaling pathways can be used to verify whether a drug has bone-promoting activity [29]. At present, studies have shown that oxygen tension and VEGF family growth factors are the main factors promoting intraarticular angiogenesis, which not only affect endothelial progenitor cells (EPCs) and osteocyte lineage but also prove that the process of angiogenesis and bone formation is coupled [30]. The VEGF family mainly includes VEGF signal transduction, HIF, MMPs and ROCK1. CD31 is also known as a platelet endothelial cell adhesion factor. It is often located in vascular endothelium, platelets and other tissue cells. It is used to mark vascular endothelium in laboratory and diagnosis. The higher the content of CD31, the more angiogenesis there is [31]. In this study, we used PCR to detect the expression of genes related to osteogenesis and angiogenesis, and we used western blotting and IHC to detect the expression levels of corresponding proteins. The results showed that SAL coating could significantly promote the expression of genes and proteins related to osteogenesis and angiogenesis.

In summary, Ti coated with SAL was prepared by MAO and PDA. The surface structure of the SAL coating was compact, and SAL was successfully loaded onto Ti by SEM and EDS. The drug release rate test showed that SAL was released slowly in the membrane and had good safety. The study also further confirmed that the SAL coating could promote bone regeneration and angiogenesis in vitro. At the same time, the promotion of bone regeneration and angiogenesis by the SAL coating in vivo was confirmed. Finally, the mechanism of the SAL coating promoting bone regeneration and angiogenesis was preliminarily discussed. SAL’s synergy with the Ti-PDA coating to promote osteogenesis and angiogenesis was successfully confirmed.

## Conclusion

In this study, the traditional Chinese medicine monomer SAL was successfully loaded onto Ti tablets by MAO and PDA. The surface morphology and element distribution of the SAL coating were observed by SEM and EDS. The results showed that the surfaces of the specimens had a microporous structure with similar average pore size. EDS and EDS mapping analysis showed that SAL could be loaded onto the surface of a MAO coating by PDA carrier. The SAL release experiment showed that SAL could be released slowly and stably from the Ti coating without the risk of sudden release. Furthermore, phalloidin and DAPI staining, Alizarin Red staining and ALP activity assay showed that the SAL coating could promote the osteogenic activity of MC3T3-E1 cells. Cell migration and tube formation experiments showed that the SAL coating could promote angiogenesis in HUVECs. The results of PCR and western blotting showed that the SAL coating could promote the expression of osteogenesis related genes and proteins RUNX2, OCN and ALP as well as angiogenesis related genes and proteins HIF-1α, MMP-2, ROCK1 and VEGF. The effects of the SAL coating on osteogenesis and angiogenesis in vivo were detected on a rat femoral defect model. Micro CT scans showed that the SAL coating could improve the integration effect of Ti implants with the surrounding defect area and accelerate the healing ability of the implants. HE and IHC staining were performed in the area around the bone defect. The distribution of trabeculae and marrow cavity in the SAL group was more regular, and the shape and density of trabeculae were better. At the same time, the expression levels of COL-1 and CD31 were higher. Goldner's trichrome analysis and Safranin O-fast green staining showed that 2 mg SAL-PDA group had more new bone tissue around the implantation site. The SAL coating could reduce the levels of TNF-α and IL-6 in vivo and had anti-inflammatory effects. In conclusion, an SAL coating on Ti can promote bone regeneration and neovascularization. SAL synergistically promotes the osteogenesis and angiogenesis of medical Ti materials.

## Data Availability

All the data and materials in this work are available in the manuscript.

## Abbreviations

Ti: Titanium
MAO: Microarc oxidation
SAL: Salidroside
PDA: Polydopamine
HE: Hematoxylin and eosin staining
IHC: Immunohistochemistry
qRT-PCR: Quantitative real-time polymerase chain reaction
PVDF: Polyvinylidene fluoride membrane
DFO: Deferoxamine
